# A systematic review of indocyanine green lymphography imaging for the diagnosis of primary lymphoedema

**DOI:** 10.1093/bjr/tqaf006

**Published:** 2025-01-21

**Authors:** Greta Brezgyte, Mike Mills, Malou van Zanten, Kristiana Gordon, Peter S Mortimer, Pia Ostergaard

**Affiliations:** School of Health & Medical Sciences, City St George’s, University of London, Cranmer Terrace, London SW17 0RE, United Kingdom; School of Health & Medical Sciences, City St George’s, University of London, Cranmer Terrace, London SW17 0RE, United Kingdom; School of Health & Medical Sciences, City St George’s, University of London, Cranmer Terrace, London SW17 0RE, United Kingdom; School of Health & Medical Sciences, City St George’s, University of London, Cranmer Terrace, London SW17 0RE, United Kingdom; Lymphovascular Medicine, Dermatology Department, St George's University Hospitals NHS Foundation Trust, London SW17 0QT, United Kingdom; School of Health & Medical Sciences, City St George’s, University of London, Cranmer Terrace, London SW17 0RE, United Kingdom; Lymphovascular Medicine, Dermatology Department, St George's University Hospitals NHS Foundation Trust, London SW17 0QT, United Kingdom; School of Health & Medical Sciences, City St George’s, University of London, Cranmer Terrace, London SW17 0RE, United Kingdom

**Keywords:** indocyanine green lymphography (ICGL), primary lymphoedema, lower limb, near-infrared fluorescence (NIRF), lymphatic system, superficial imaging, indocyanine green (ICG)

## Abstract

**Objectives:**

This systematic review aims to evaluate the use of indocyanine green lymphography (ICGL) for the investigation of the lymphatics in the lower limbs of primary lymphoedema patients.

**Methods:**

MEDLINE and EMBASE articles from January 1, 2000 to September 1, 2023 were searched for. A total of 11 studies were included in the review after a two-stage screening process.

**Results:**

Data on patient demographics, ICG contrast injection technique, imaging protocols, and imaging outcomes were summarized and reviewed in detail. The review highlights the lack of commonality in protocols used. Factors important for good imaging are highly variable, particularly the number of injections, their location, and whether they are delivered intradermally or subcutaneously.

**Conclusions:**

ICGL has strong potential to become a diagnostic tool to diagnose lymphoedema due to its non-ionizing nature and cost-effectiveness. However, due to the lack of thorough phenotyping and genotyping of patients included in the studies, uncertainty still exists as to the value of the described imaging features such as splash, starburst, and diffuse dermal rerouting patterns. Future studies, therefore, should aim to explore the diagnostic utility of ICGL for lymphoedema further through the imaging of primary lymphoedema patients with a confirmed genetic diagnosis and using standardized imaging protocols.

**Advances in knowledge:**

ICGL is a strong candidate for advancing the diagnosis and understanding of primary lymphoedema, and monitoring response to treatment, but protocol heterogeneity and a lack of consistency in reporting imaging details and patient phenotyping currently hold it back.

## Introduction

Lymphoedema is a condition of chronic swelling due to a compromized lymphatic system. Affecting over 66 million people worldwide, there are currently no cures, and treatments aim only to reduce swelling.^1^^,^^2^ This lack of therapeutic options is partly due to the paucity of knowledge regarding the function and anatomy of human lymphatics, despite its importance for regulating fluid balance, preventing infection, and involvement in conditions ranging from cancer to obesity.^3^^,^^4^

Lymphoscintigraphy is the most used imaging technique for diagnosing lymphoedema, offering reliable assessments of lymphatic function.^5^^,^^6^ Lymphoscintigraphy is limited however by poor image quality.^7^ Single Photon Emission CT, in combination with X-ray CT, has also been employed to image lymph nodes due the enhanced anatomical detail and the ability to generate 3D images.^7^^,^^8^ With the injection of a suitable contrast material, CT alone is capable of providing high-resolution images of lymphatic vessels.^8^ However, each of these techniques is limited by the associated exposure to ionizing radiation. In contrast, Magnetic Resonance lymphangiography is a non-ionizing alternative, employed either with or without the use of an exogenous contrast agent, providing reasonable spatial resolution. However, it does not enable real-time visualization of lymphatic flow.^9^^,^^10^

Indocyanine green (ICG) lymphography (ICGL) meanwhile facilitates non-ionizing, real-time lymphatic imaging *in vivo*^11^ and has been used to aid sentinel lymph node biopsy for cancer management,^12^ identify lymphatics suitable for lymphovenous anastomosis surgery,^13^ and to investigate the effectiveness of manual lymphatic drainage (MLD).^14^ ICGL does not seem to cause lymphatic inflammation or vessel damage,^15^ and is hence gaining traction as both a research and clinical tool. ICG has infrared fluorescent properties, which are therefore rapidly attenuated within only a few centimetres below the skin surface,^16^ an obstacle in patients whose subcutaneous tissue has thickened.^17^ Like other 2D imaging techniques, including lymphoscintigraphy, lymphatic vessel depth can also not be obtained,^18^ but high spatiotemporal resolution visualization of superficial lymphatic vessels is possible.

Lymphoedema is either primary (PL), due to an intrinsic fault (presumed genetic),^19^ or caused by extrinsic damage (secondary), for example, lymph node removal.^20^ The discovery of gene mutations causing lymphatic anomalies has revealed different mechanisms that disturb lymph drainage in PL.^21^ Improved management of PL will require definitive imaging of the lymphatic system to identify the pathological mechanisms at play and categorize the lymphatic fault before intervention. ICGL is a potential low-cost, non-ionizing candidate for this.

In this study, we comprehensively review the literature describing ICGL in the lower limbs in the context of PL and highlight its diagnostic potential.

## Methods

### Paper identification

This systematic review was conducted according to the Preferred Reporting Items for Systematic Reviews and Meta-Analyses (PRISMA) guidelines.^22^ A comprehensive search of the ICGL literature was conducted, retrieving Medline and Embase records published between January 1, 2000 and September 1, 2023. Search terms: Diagnos* imag* OR Diagnos* tool* OR Diagnos* method* OR Diagnos* technique* OR Lymphography OR Diagnostic Imaging AND Primary Lymphoedema OR Congen* Lymphoedema OR Lymphan* OR Lymph* malf* AND Indocyanine green OR ICG OR Indocyanine OR Indocyanine Green OR Fluoresc* OR NIRF OR Near-infrared were used and duplicated articles removed.

### Screening stage 1

Abstracts were screened using the inclusion criteria summarized in Table 1. Conference abstracts/reports, reviews, letters/replies, book chapters, and single case studies were excluded, as were abstracts not mentioning ICG imaging and lymphoedema or related terms. Abstracts referencing the use of animal or cadaveric subjects were also removed.

**Table 1.** List of inclusion criteria applied in this systematic review.

Inclusion criteria	
1.	Records including ICG imaging in primary lymphoedema.
2.	Human studies only.
3.	Presentation of lower limb ICGL findings with descriptions.
4.	The imaging method was described by the authors.
5.	Manuscripts from January 1, 2000 to September 1, 2023.

Abbreviation: ICG = indocyanine green; ICGL = indocyanine green lymphography.

### Screening stage 2

Full texts were then obtained. Further single case reports and papers not reporting original ICGL data or detailed imaging methods were removed, as were studies of healthy controls and/or secondary lymphoedema cases only. The remaining papers were analysed independently by 2 assessors (G.B. and P.O.), and only papers describing detailed imaging methods for the purpose of diagnosing lower limb primary lymphoedema were retained. Papers using ICGL for other purposes (eg, interoperative ICG imaging used during lymphatic surgery) were excluded.

## Results

### Study inclusion

**Figure 1.**
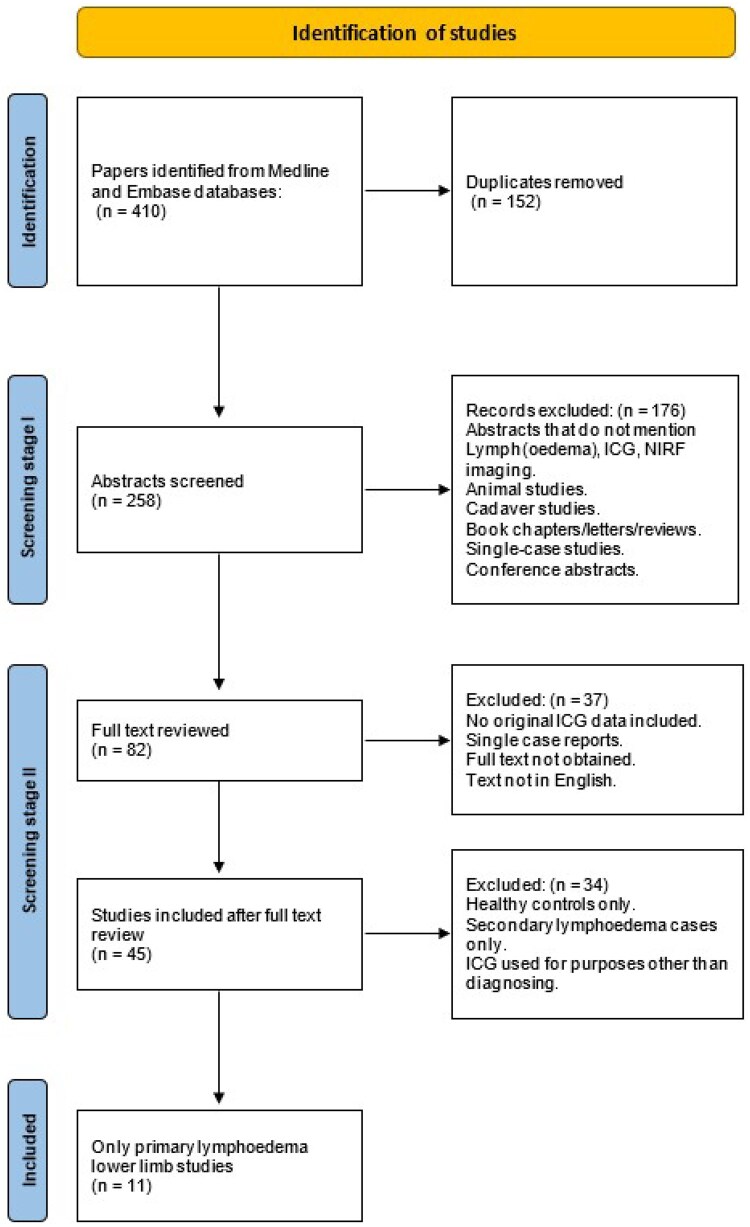
Study selection flow chart. Medline and Embase databases revealed a total of 410 sources. After the application of inclusion and exclusion criteria, a total of 11 articles were shortlisted for this review. Note that some single case reports were removed in screening stage, 2, as it was only after full-text retrieval that it became clear the article reported only 1 case.

The initial Medline and Embase searches yielded 410 records (Figure 1). After duplicate removal, 258 abstracts were reviewed, of which 82 were retained after having passed screening stage 1. After full text review, 11 studies focusing on ICGL of the lower limbs in PL were included in this systematic review (Table 2). For these studies, data on patient recruitment, diagnosis, ICG contrast injection, imaging protocol, and imaging outcomes are presented.

**Table 2.** Overview of papers shortlisted in the systematic review including a summary of the number of individuals included in the studies.

Reference	Female	Male	Age range (years)	Average age (years)	Age of onset (average age years)	Primary lymphoedema (number of individuals)	Secondary lymphoedema (number of individuals)	Limbs imaged in the primary lymphoedema cases
Akita et al., 2013	115	19	9-82	58.5	ns	39	95	Lower and upper limbs
Gentili et al., 2021	26	6	18-73	38	ns	6	26	Lower limbs
Hara and Mihara, 2020	96	7	11-82	57.8	ns	10	93	Lower limbs
Mackie et al., 2022	ns	ns	ns	ns	ns	88	478	Lower and upper limbs
Mangialardi et al., 2020	19	1	ns	43.4	14-70	20	0	Lower and upper limbs
Matsumoto et al., 2019	59	4	20-78	56	ns	6	55	Lower limbs
Pons et al., 2019	77	5	ns	45.5	ns	21	61	Lower and upper limbs
Suami et al., 2022	215	63	ns	47.1^a^	ns	112	166	Lower limbs
Yamamoto et al., 2015	20	11	12-82	42.5	0-78 (28)	31	0	Lower limbs
Yoshida et al., 2020^25^	48	26	33-95	73.6	25-93 (68)	74	0	Lower limbs
Yoshida et al., 2020^24^	35	21	33-95	73.1	25-93 (68)	56^b^	0	Lower limbs

a Average age for primary lymphoedema cases only.

b Suspected a subset of Yoshida et al. (2020).^25^

Abbreviation: ns = not specified.

### Patient cohorts

Among the 11 studies, 4 enrolled patients with PL only.^23–26^ The remainder included patients with primary and secondary lymphoedema.^27–33^ Number of cases, their age and sex, and limbs imaged are summarized in Table 2.

### ICG injection protocol

The most commonly used fluorescent agent (7/11) was Diagnogreen (Table 3). Verdye was used in 2 studies, 1 study used ICC-Pulsion and another did not specify. Some diluted the ICG in saline^31^^,^^33^ or water,^23^ but most did not specify. ICG agents were administered at 0.5% (6/11) or 0.25% (4/11) concentrations, or not reported.

**Table 3.** Summary of ICG contrast injection protocols.

Reference	ICG manufacturer	Concentration (%)	Volume per injection (mL)	Injection plane
Akita et al., 2013	ns	ns	0.3	Subcutaneous
Gentili et al., 2021	Diagnogreen	0.5	0.2-0.3	Subcutaneous
Hara and Mihara, 2020	Diagnogreen	0.5	0.05	Subcutaneous^c^
Mackie et al., 2022	Verdye	0.5^a^	0.05-0.1	Intradermal^c^
Mangialardi et al., 2020	ICC-Pulsion	0.5	0.2-1	Intradermal
Matsumoto et al., 2019	Diagnogreen	0.5	0.05	Intradermal
Pons et al., 2019	Diagnogreen	0.5	0.2-0.4	Subcutaneous
Suami et al., 2022	Verdye	0.25^b^	0.05-0.1	Intradermal
Yamamoto et al., 2015	Diagnogreen	0.25	0.2	Subcutaneous
Yoshida et al., 2020^25^	Diagnogreen	0.25	0.2	Subcutaneous
Yoshida et al., 2020^24^	Diagnogreen	0.25	0.2	Subcutaneous

a 25 mg Verdye mixed with 5 mL saline.^34^

b 25 mg Verdye mixed with 10 mL saline.^33^

c Protocol based on previous publication by the authors.

Abbreviation: ICG = indocyanine green; ns = not specified.

All studies reported the volume of ICG fluorescent agent injected per site, ranging from 0.05 to 1 mL. In 5, the administered volume varied between participants. Over half (7/11) injected the agent subcutaneously, the others intradermally. Some studies mentioned the use of local numbing with lidocaine,^29^ xylocaine,^23^ or a topical cryogenic numbing device.^31^^,^^33^

### Injection sites

**Figure 2.**
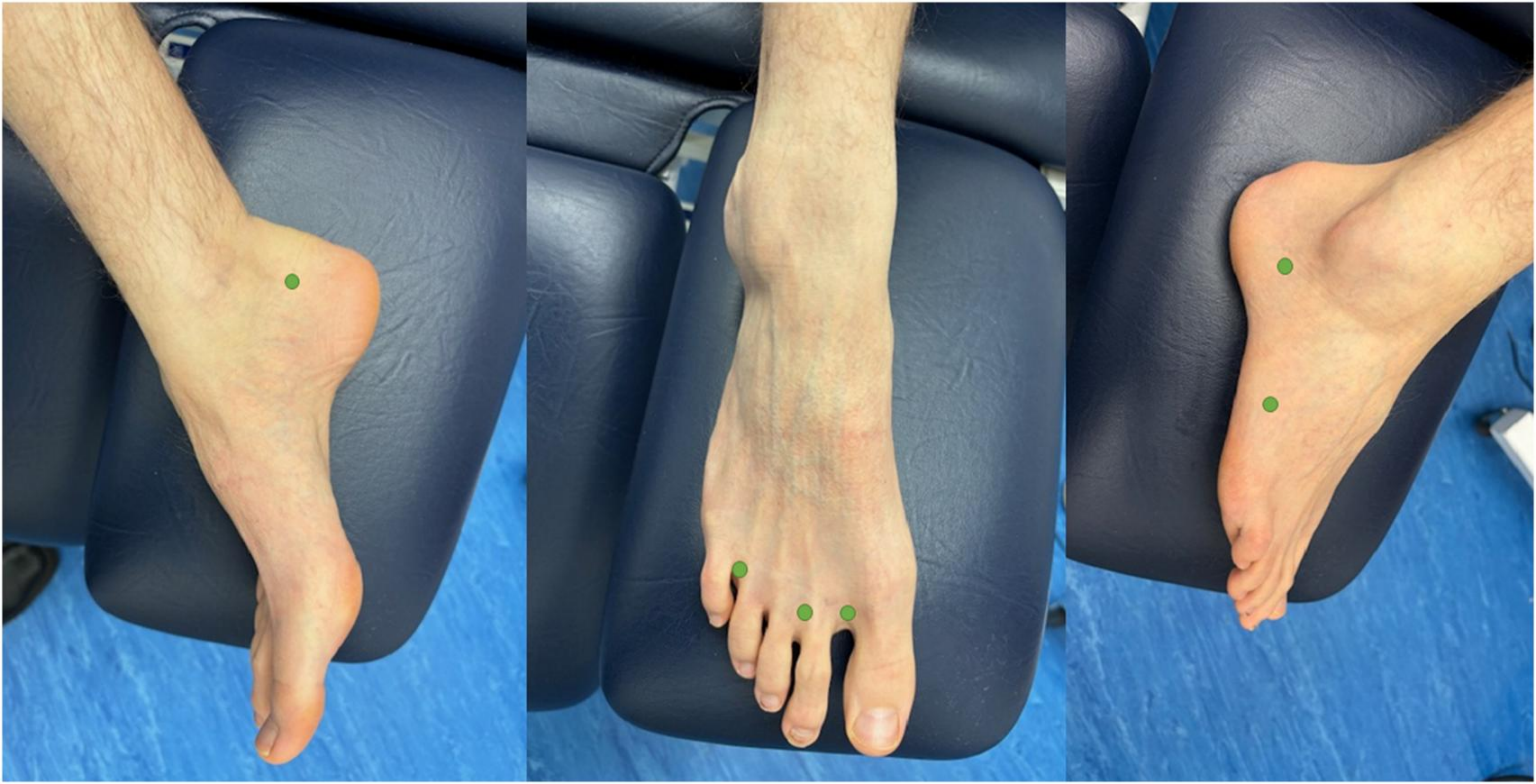
The 11 studies use a combination of 6 sites for ICG contrast injections in the foot, marked here with a green dot. (A) Shows the medial side of the foot, (B) shows injections in the forefoot, and (C) shows the lateral aspect of the foot indicating midfoot and rearfoot injections.

Between 1 and 4 injection sites per foot were employed, including at least one of the web spaces of the toes (Table 4). Of the 6 sites used (Figure 2), the most common was the first web space of the toes (8/11). Four studies injected into the second and/or fourth web spaces. The second most common site was laterally, towards the rearfoot near the lateral malleolus and Achilles tendon. One study reported an injection into the lateral side of the superior edge of the knee in addition to 2 foot injections.^30^

**Table 4.** Summary of injection sites in the feet for imaging of the lower limbs.

Reference	Total number of injections	Web space of the toes				The lateral aspect of the foot		Medial aspect	Other injection sites
		First	Second	Third	Fourth	Midfoot	Towards rearfoot		
Akita et al., 2013	1	×							
Gentili et al., 2021	2		×		×				
Hara and Mihara, 2020	3	×					×		Lateral side of the superior edge of the knee
Mackie et al., 2022	4	×				×	×	Below medial malleoli	
Mangialardi et al., 2020	2		×				Border of AT		
Matsumoto et al., 2019	4	×			×	×	Posterior side of the ankle		
Pons et al., 2019	2		×		×				
Suami et al., 2022	4	×				×	×	Below medial malleoli	
Yamamoto et al., 2015	2	×					Border of AT		
Yoshida et al., 2020^25^	2	×					Border of AT		
Yoshida et al., 2020^24^	2	×					Border of AT		

Abbreviation: AT = Achilles tendon.

### Imaging protocols

Most commonly (8/11), imaging commenced immediately after ICG contrast injection (Table 5). The duration of imaging was not consistently reported. One study reported the exam lasts 10-15 min,^29^ whilst others reported ∼1 h.^31^^,^^33^ Two studies repeated imaging after 2 h, while 3 studies reimaged patients after 6-24 h.

**Table 5.** Summary of imaging protocols.

Reference	Initial imaging time after injection	Repeat imaging time after initial contrast injection	Near infrared detector camera
Akita et al., 2013	1 h	2 h	PDE
Gentili et al., 2021	Immediately	None reported	PDE
Hara and Mihara, 2020	Immediately	2 h	PDE
Mackie et al., 2022	Immediately	None reported	PDE Neo II
Mangialardi et al., 2020	12-18 h	None reported	PDE
Matsumoto et al., 2019	Immediately	6 times after a 5-min exercise period^a^	PDE
Pons et al., 2019	ns	None reported	PDE
Suami et al., 2022	Immediately	10 min, during manual lymphatic drainage	PDE Neo II
Yamamoto et al., 2015	Immediately	12-18 h	PDE
Yoshida et al., 2020^25^	Immediately	6-24 h	PDE^b^
Yoshida et al., 2020^24^	Immediately	12-18 h	PDE

a Each additional imaging session was carried out after 5 min of treadmill (2 km/h) exercise with a total of 30 min exercise per imaging session.

b Based on previous publication by the authors.

Abbreviation: h = hours; min = minutes; ns = not specified; PDE = photodynamic eye.

Only a few studies disclosed the position of the patient (standing, supine, lateral, or prone) when imaged,^25^^,^^27^^,^^29^^,^^33^ and, when imaging both limbs, if they were injected and imaged simultaneously.^23^^,^^27–29^^,^^33^

Lymph flow is often delayed at the ankle joint,^25^ and exercise or massage is considered to purposefully encourage ICG contrast uptake. Some have described improved visualization of lymphatic pathways after 30 min of manual lymphatic stimulation in secondary lymphoedema upper limb imaging.^35^ Of the papers reviewed, some employed toe and ankle flexions,^25^ while others experimented with exercise on a treadmill to improve uptake of ICG contrast^27^ and thus reduce imaging time. Two studies reported checking for spontaneous movement of ICG contrast immediately after injection, then after 10 min, the imaging would continue while MLD was performed.^31^^,^^33^ It was suggested that the application of ICG contrast-guided MLD reduces imaging time.^33^

### Imaging features and outcome measures

#### Functional lymphatic vessels

**Figure 3.**
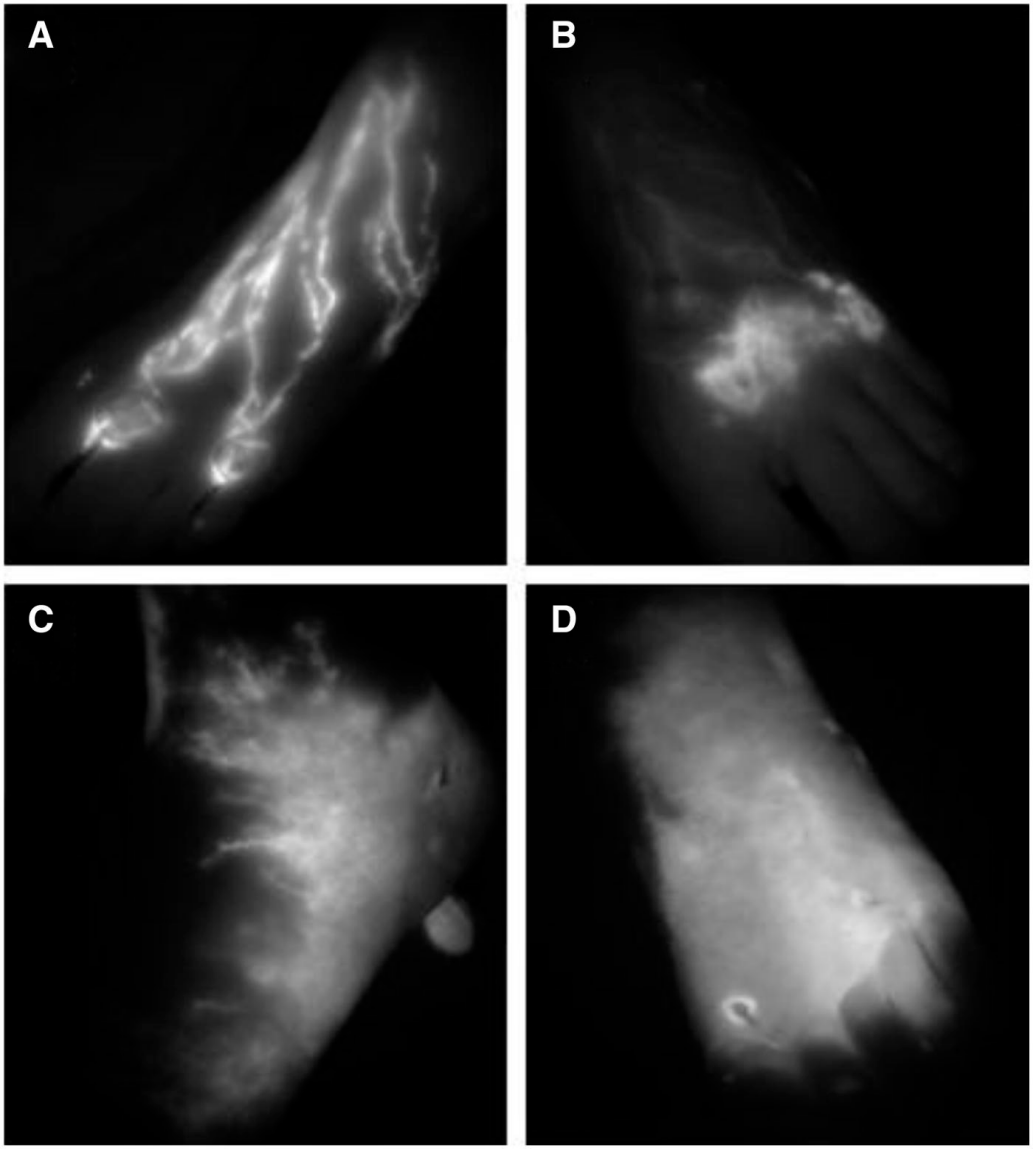
Lymphography patterns observed with the near infrared detector camera following the definitions of Yamamoto et al.^37^ (A) Linear, the normal superficial lymphatic pattern; and the abnormal lymphatic patterns: (B) splash; (C) stardust; and (D) diffuse, collectively called dermal backflow patterns and indicate greater disease severity in order of appearance (from B to D). (Images from lower limb ICGL in primary lymphoedema shared by City St George’s Lymphovascular Research Group.)

All studies agree that in healthy limbs, lymphatic vessels appear on ICGL as a linear pattern spreading from the injection site (Figure 3A) towards the groin. Interestingly, one article reported a third of “unaffected” clinically healthy limbs in patients with unilateral lymphoedema showed abnormality on ICGL,^23^ and age-related declines in lymphatic function were demonstrated.^24^

#### Retrograde flow in the collector vessels

Transport of lymph should be a one-directional flow from absorbing initial lymphatics through ever enlarging lymphatic vessels to lymph nodes. Larger main limb lymphatics or collecting vessels ensure flow against gravity due to lymphatic contractility and lymphatic valves. If they fail, retrograde or reverse lymph flow can result. One study, recording the presence of valves and the direction of flow, was able to assess collector vessel function. Faulty contractility and retrograde or reverse flow across incompetent valves could be imaged and recorded by ICGL.^31^

#### Dermal backflow

Any hold-up of downstream flow can result in reverse or retrograde flow of lymph back towards initial lymphatics in the dermis. This is dermal backflow (DB) and is a diagnostic sign of lymphoedema. Visible on ICGL as a vessel network within the skin extending well beyond the injection sites, DB can be seen as soon as 4-5 min after contrast injection^29^ and can spread and mask underlying vessels.^30^ Generally, the more extensive the DB, the more severe the disruption to limb lymph flow and so the severity of lymphoedema. In addition to the retrograde filling of dermal lymphatic vessels, similar appearances on ICGL may also be due to the diffusion of ICG out of the lymphatic vessels into the interstitial tissues.^36^

Dermal backflow in lymphoedema has been grouped into 3 different patterns: “splash”, “stardust”, and “diffuse” (Figure 3B-D),^37^ and these definitions were adopted by some of the studies (Table 6), while other authors simply report the prescence of DB. Others use definitions like “distal” or “proximal” DB to define its location, and “less enhancement” or “no enhancement” to convey a degree of vessel hypoplasia or aplasia.^23–26^ There were no significant sex-related differences reported between the different lymphography patterns.^24^

**Table 6.** ICG imaging features and outcome measures used to evaluate lymphoedema.

Reference	Linear	Dermal backflow^a^			Other dermal backflow definitions	Time to groin	Other types of measures
		Splash	Stardust	Diffuse			
Akita et al., 2013		×	×	×			
Gentili et al., 2021					×		
Hara and Mihara, 2020	×				×		% of linear pattern
Mackie et al., 2022					×		Retrograde flow, patent vessels, contractility
Mangialardi et al., 2020	×		×	×	NE, LE, dDB, PDB		
Matsumoto et al., 2019	×				×	Yes	Dermal backflow appearance rate
Pons et al., 2019	×	×	×				Collateral vessels
Suami et al., 2022					×		Compensatory drainage regions
Yamamoto et al., 2015	×				NE, LE, dDB, PDB		
Yoshida et al., 2020^25^	×				LE, dDB, eDB	Yes	
Yoshida et al., 2020^24^	×				LE, dDB, eDB	Yes	

Abbreviations: dDB = distal dermal backflow; eDB = extended dermal backflow; ICG = indocyanine green; LE = low enhancement; NE = no enhancement; PDB = proximal dermal backflow (similar to eDB).

a As defined by Yamamoto et al 2011.^37^

#### Transport capacity

Some studies offered measures of transport capacity such as time to groin (Table 6): the time taken to visualize the inguinal nodes after ICG injection.^24^^,^^25^^,^^27^ In healthy limbs, the superficial inguinal lymph nodes may be observed within 10-15 min, becoming more delayed as lymphoedema worsens.^24^^,^^25^^,^^28^ However, with exercise, these could also be observed after 15 min in lymphoedema patients.^27^

#### ICG contrast distribution

In non-lymphoedematous limbs, drainage of ICG contrast appears to follow predictable routes based on the location of injection.^38^ Alternative drainage routes may appear as a result of lymphoedema^32^^,^^33^; however, a particular pattern (labelled the “print sign”) was observed in some PL cases where signal distal to the injection site on the foot plantar surface and plantar and dorsal surface of the toes was recorded. The authors suggest this feature could be of diagnostic utility.^23^

## Discussion

Lymphoscintigraphy has shown use in phenotyping and improving understanding of the causal mechanisms of primary lymphoedema.^39^ The objective of this review was to explore whether ICGL has been used for this purpose or what features may be useful in this regard. We limited our investigation to imaging in the lower limbs of PL patients (the most commonly swollen region), and this review provides evidence that ICGL of lymphatic vessels is capable of demonstrating altered flow dynamics and drainage in these cases.

### Protocol standardization

This systematic review shows that ICGL protocols are variable; including the ICG agent used, the concentrations or volumes administered, and injection sites and depth.

#### ICG agent

The most commonly used manufacturer of ICG was Diagnogreen. Reasons for this were not clearly described but are usually related to local availability and cost. ICG is commonly provided in a sterile powder and administered diluted with water or saline and local anaesthetic to reduce discomfort of injection. Though saline has been used in some studies reported here, anecdotal concerns regarding ICG solubility and spectral characteristics have been raised, and dilution with water may be preferable. The concentration and volume per injection were found to vary substantially. In a study conducted by Visconti et al. (2017)^40^, it was reported that diluting ICG powder (ICC-Pulsion, Pulsion Medical System, Germany) with 5 mL of sterile water containing 0.5% xylocaine significantly reduces the pain associated with intradermal injections in patients with primary and secondary lymphoedema.

To what extent these impact performance could not be determined; however, that administered concentration influences the fluorescent properties of ICG is known.^41^ Future studies investigating the optimal conditions of ICG contrast solution and dilution would be beneficial in humans, as has been done in animals.^42^^,^^43^

#### Anatomical injection sites

Shinaoka et al.^38^ studied lymphatic drainage routes in non-lymphoedematous cadavers with 19 injections in the foot, allowing them to classify 4 distinct lymphatic drainage routes: anteromedial, anterolateral, posteromedial, and posterolateral. It is suggested that in addition to injecting into the web space between the toes, injection sites in the medial, lateral, and posterior aspects of the foot are also needed for full evaluation of all the lymphatic pathways to improve our understanding of leg lymphoedema. The number of injections varied across the 11 studies, with only 2 studies^31^^,^^33^ covering all 4 main drainage routes. This suggests that studies only using injection in the web spaces between the toes could fail to visualize some of the lymph drainage pathways. Thus, interpretation and comparison of results from ICGL studies need to consider this.

#### Injection depth

A mix of subcutaneous and intradermal injections were employed in the reviewed studies. Sub-epidermal injections (high dermis) for lymphoscintigraphy result in faster lymphatic uptake and flow^44^ but for quantitative results, for example, lymph node uptake and limb lymph drainage function, subcutaneous injections appears better.^45^ To what extent this is the same for ICGL is not known but in theory access and uptake to superficial lymphatics ought to be better with intradermal injections. Only 4 of the studies used intradermal injections.

#### Time of imaging

In addition to the injection sites and depth, timing of imaging is essential. Some carried out repeat images 6-24 h post injection, however it has been reported that repeat imaging 24 h post injection is comparable to earlier imaging.^46^ It is also important to note that, over time, DB may arrise which masks underlying vessels and so prevent a more complete picture of the lymphatic network.^30^

### Interpretation of imaging

#### DB (reflux) patterns

Despite the heterogeneity of ICGL protocols used, all studies were able to visualize lymphatic vessel and DB patterns. The linear lymphatic pattern was the most reported structural finding and is thought to represent the normal superficial lymphatic network, as evidenced by healthy controls displaying this pattern.^47^ Different backflow patterns from “splash,” “stardust,” to “diffuse” are suggested to grade the severity of disease,^37^ and some studies also tried to classify the DB by location (distal vs proximal). However, no clear classification linking these to specific PL phenotypes has been attempted. Regardless of the definitions utilized, it will be interesting in future studies to see how these can be used to categorize different phenotypic or genotypic forms of PL.

#### Retrograde flow in collector vessels

Dysfunction in the lymph-collecting vessels resulting in a reversal of lymphatic flow has been described commonly in the literature as a pathological feature of primary lymphoedema especially in patients with lymphoedema distichiasis syndrome.^48^ Contrary to the accepted knowledge of the pathological alterations in PL, retrograde lymph flow with valve incompetence in the lymphatic vessels was rarely reported in the selected studies. However, only 1 study included a method for the observation of retrograde flow, which they observed in 2 patients with confirmed lymphoedema distichiasis syndrome, and they discussed valve incompetence as a potential feature to diagnose lymphoedema.^31^ Thus, exploring ICGL utility according to lymphoedema pathogenesis and analysing the signature imaging features for each genotype could establish retrograde lymph flow analysis as a useful diagnostic measure in ICGL in combination with the clinical presentation.

#### Flow of lymph

Other measures for lymphatic function could relate to the speed with which the ICG gets transported or lymphatic contractility. In 2010, Unno et al. estimated lymphatic pumping pressure in human subjects with ICGL via the application of pressure cuffs to occlude lymphatic vessels.^49^ Shortly thereafter, ICGL was used to measure lymphatic contractile frequency and the speed of ICG contrast boluses in the vessels.^50^^,^^51^ Pumping frequency was also reported in an ICGL study of rats following lymph node removal and X-ray irradiation, showing increased and more erratic lymphatic pumping following lymphatic injury.^52^ None of the 11 studies attempted measurements as detailed as these, but a few investigated the time taken for the ICG contrast to reach the groin. For this to be a useful tool and to allow comparison between individuals or between studies, the protocol needs standardizing, particularly regarding exercise, which can greatly influence the speed.^24^^,^^25^^,^^27^ It should also be noted that these measures of speed relate only to transport of ICG via the superficial lymphatics, which are detectable with ICGL.

#### Diagnostic utility

The ability of ICGL to demonstrate abnormal lymphatic vasculature was clearly demonstrated within this review, and ICGL has also been shown sensitive enough to detect subtle lymphatic anomalies prior to clinical signs of lymphoedema.^14^ In all 11 articles, patients were reported as presenting with swelling prior to the described imaging. The ability to diagnose lymphoedema in the absence of evident disease was not explored, though some reported ICGL was used to confirm the lymphoedema diagnosis.^24^^,^^25^

#### Phenotyping through imaging

With the established imaging patterns and methods for assessment of lymph transport, ICGL could possibly aid phenotyping of primary lymphoedema. However, there is little published on this. The 11 studies in this review included over 460 reported cases of PL, but only 2 papers specified the type. One reported the inclusion of 2 lymphoedema distichiasis cases,^31^ and the other listed 11 cases with genetic variants identified in known PL genes; however, causality was not confirmed.^23^

Some case studies, excluded from the systematic review, used ICGL to confirm the presence or absence of lymphoedema in genotyped family members^53^^,^^54^; however, the reports included too few cases to enable any meaningful genotype-phenotype correlations. Thus, there are no studies in the literature that systematically look at genotyped PL cases with ICGL to determine the pathology.

Based on a previous lymphoscintigraphy study, clear phenotypic differences between Milroy disease and lymphoedema distichiasis syndrome were demonstrated on imaging.^39^ We believe ICGL can be used in similar ways to define genetic groups. However, if the studies do not genotype, or as a minimum thoroughly describe the phenotypic details of their patients, then the ICGL can only distinguish whether a patient has lymphoedema or not.

## Conclusion

Depending on the outcome measures of interest, ICGL seems to be a suitable tool for visualizing lymphatic vessels and could prove useful for deep phenotyping of primary lymphoedema phenotypes. There is a clear lack of consensus in injection protocols, particularly regarding anatomical injection sites that will greatly affect which superficial lymphatic pathways can be visualized with ICGL. Robust outcome measures, that is consensus on criteria for determining lymphatic abnormalities, are also lacking. This limits the current utility of ICGL for the diagnosis of lymphoedema. The proposal for injections that will allow ICG contrast to reach each of the 4 main lymphatic drainage pathways is recommended.^38^ The depth of injection influences lymphatic access and also needs careful consideration. Future research should look at optimizing and implementing the best ICGL imaging protocols and developing a range of objective measures for quantifying and subjective measures for describing imaging features. Studies applying this technique for phenotyping primary lymphoedema patients could also then be explored.
